# Genomic analyses indicate the North American Ap-ha variant of the tick-vectored bacterium *Anaplasma phagocytophilum* was introduced from Europe

**DOI:** 10.1186/s13071-023-05914-x

**Published:** 2023-08-28

**Authors:** Matthew L. Aardema

**Affiliations:** 1 Department of Biology, Montclair State University, Montclair, NJ USA; 2 Institute for Comparative Genomics, American Museum of Natural History, New York, NY USA

**Keywords:** *Ixodes*, Genetic bottleneck, Founder event, Anaplasmataceae, Phylogeography

## Abstract

**Background:**

*Anaplasma phagocytophilum* is a tick-vectored, obligately intracellular bacterium that infects a diversity of vertebrate hosts. In North America, the Ap-ha variant of *A. phagocytophilum* can cause dangerous infections in humans, whereas symptomatic human infections in Europe are rare. Conversely, the European host-generalist ecotype of *A. phagocytophilum* frequently causes illness in domestic ruminants while no comparable infections have been recorded from North America. Despite these differences in pathogenicity, the Ap-ha variant is closely aligned phylogenetically with the European host-generalist ecotype. Furthermore, North American populations of *A. phagocytophilum* are less genetically diverse than those in Europe. Taken together, these observations suggest that the North American Ap-ha variant may represent an introduced population of this bacterium.

**Methods:**

Data from publicly available whole genomes of *A. phagocytophilum* were used to compare phylogeographic patterns and the extent of genetic divergence between the North American Ap-ha variant and the European host-generalist ecotype.

**Results:**

The results confirm that North American Ap-ha samples are phylogenetically nested within the diversity of the European host-generalist ecotype, and that Ap-ha likely radiated within the last 100 years. As expected, the Ap-ha variant also exhibited relatively low genetic diversity levels compared to the European host-generalist ecotype. Finally, North American Ap-ha harbored significantly more derived alleles than the European host-generalist *A. phagocytophilum* population.

**Conclusions:**

Collectively, these results support the hypothesis that the Ap-ha variant was recently introduced to North America from Europe and underwent a strong genetic bottleneck during this process (i.e. a ‘founder event’). Adaptation to novel vectors may have also played a role in shaping genetic diversity and divergence patterns in these pathogenic bacteria. These findings have implications for future studies aimed at understanding evolutionary patterns and pathogenicity variation within *A. phagocytophilum*.

**Graphical Abstract:**

**Figure:**
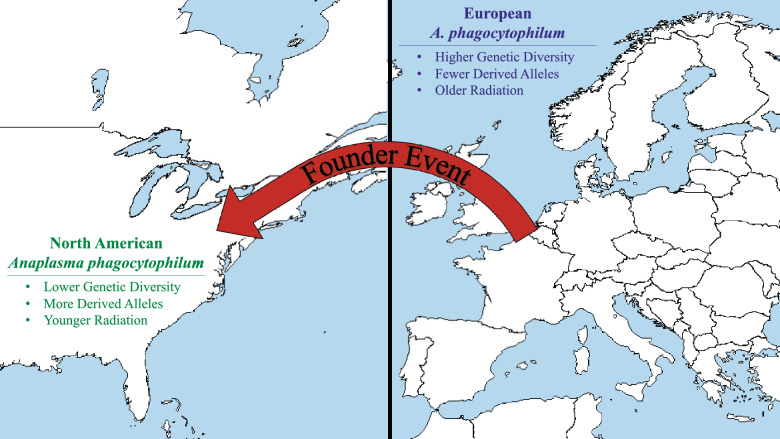

**Supplementary Information:**

The online version contains supplementary material available at 10.1186/s13071-023-05914-x.

## Background

*Anaplasma phagocytophilum* is a complex of obligately intracellular alphaproteobacteria, vectored between vertebrate hosts by hard ticks in the genus *Ixodes* [1]. Two distinct variants of *A. phagocytophilum*, ‘Ap-ha’ and ‘Ap-V1,’ are widely distributed in North America [2, 3]. Of these, the Ap-ha variant is the better characterized as this is the variant predominantly found to cause illness in humans and domestic animals such as dogs and horses. Its natural reservoirs appear to be primarily rodents [1]. In contrast, the Ap-V1 variant colonizes white tailed deer (*Odocoileus virginianus*) and has not been isolated from other mammalian hosts [2, 4, 5]. Accordingly, it is considered non-pathogenic.

In Europe, three distinct ecotypes of mammal-infecting *A. phagocytophilum* have been identified, each appearing to circulate in a distinct enzootic cycle [6, 7]. One of these ecotypes is largely limited to burrowing mammals, with bacteria being vectored by the specialist tick *Ixodes trianguliceps* [6–8]. In the other two enzootic cycles, the castor bean tick, *Ixodes ricinius*, is the main vector. One of these two ecotypes is considered a specialist, with roe deer (*Capreolus capreolus*) being the primary reservoir. The other ecotype is a host-generalist, having been isolated from a wide variety of wild and domestic mammalian hosts [6, 7, 9].

While rates of human *A. phagocytophilum* infection appear to be similar for North America and Europe, symptomatic cases are far more common in the USA compared to European countries (reviewed in [10, 11]). Conversely, illness caused by *A. phagocytophilum* infection in domestic ruminants (i.e. cattle, goats and sheep) has only been documented in Europe [1]. As there are clear continental differences in the specific species of vector and natural reservoir that comprise the enzootic cycles of *A. phagocytophilum*, genetic differences that may have arisen as the result of host adaptation in specific ecological contexts could play a role in shaping contrasting patterns of pathogenicity [1, 12, 13].

*Anaplasma phagocytophilum* from North American and Europe have been shown to exhibit a degree of genetic differentiation, particularly in fast evolving genes such as *MSP2/P44*, *groEL* and *ankA* (e.g. [3, 9, 14–16]). However, additional analyses have revealed that North American samples of the Ap-ha variant are closely aligned genetically with the European host-generalist ecotype, and in some studies samples from the USA were phylogenetically nested among samples of the European host-generalist population [6, 9, 17–21]. Collectively, examined bacteria from the North American Ap-ha variant and the European host-generalist ecotype are sister to the European roe-deer specialist ecotype [6, 9]. Other studies have shown that *A. phagocytophilum* in North America is less genetically diverse compared to European populations (e.g. [3, 11, 21, 22]). Taken together, the potential phylogenetic nesting of the North American Ap-ha variant within the European host-generalist ecotype as well as Ap-ha’s relatively low genetic diversity suggest that North American *A. phagocytophilum* may represent an introduced population from Europe [23].

Whole genome analyses have proven useful for examining patterns of divergence and evolutionary change in pathogenic bacterial species (reviewed in [24–26]), including *A. phagocytophilum* [27–32]. In this study, I used data from publicly available whole genomes of *A. phagocytophilum* to examine relatedness and the extent of genetic divergence between the North American Ap-ha variant and the European host-generalist ecotype, with a particular focus on genes found to be differentially upregulated during vector or reservoir colonization. I also explored the hypothesis that the North American *A. phagocytophilum* population was introduced from Europe and that its establishment in the Western Hemisphere was relatively recent. Finally, I examined evidence that North American *A. phagocytophilum* underwent a genetic bottleneck due to a founder event during colonization.

## Methods

### Genomic data

**Fig. 1:**
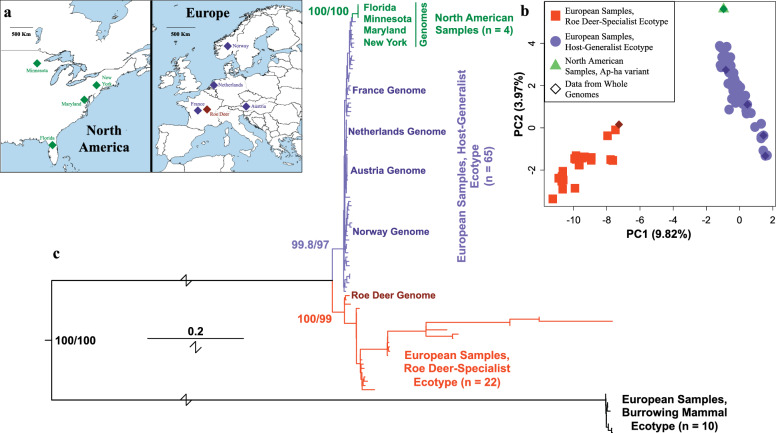
Genomic sample origins and inferred genetic relationships among the *Anaplasma phagocytophilum* samples examined. **a** The geographic locations from which the genomic data derived. The left panel shows the US states from which each of the 4 North American samples (Ap-ha variant) were isolated, and the right panel shows the countries from which the 4 European samples (host-generalist ecotype) were isolated. Also shown in the right panel is the roe-deer specialist ecotype sample, used for outgroup comparisons. This sample was isolated from France. **b** PCA of North American Ap-ha variant samples (in green), European host-generalist ecotype samples (in blue) and European roe deer-specialist ecotype samples (in red). Both axes are labeled with the percent of genetic variance explained. **c** Approximate maximum-likelihood phylogeny [37] based on multi-locus sequence typing data using unique haplotypes from Huhn et al. [6], with the addition of the orthologous sequences from the 9 focal genomes. Also included are samples from the burrowing-mammal specialist. Genome data are labeled in the figure. Colors correspond to the variant/ecotype and are the same as in the PCA. Numbers next to the nodes corresponding to divergences between ecotypes/variants indicate support values from 10,000 ultrafast bootstrap (UFBoot) replicates [40], and 10,000 replicates of the Shimodaira-Hasegawa-like approximate likelihood ratio test (SH-aLRT; [41–43]). PCA, Principal component analysis

To compare phylogeographic patterns between North American and European *A. phagocytophilum*, I leverage four previously published genomes from each of these two broad geographic regions (8 genomes in total; Fig. 1a; Additional file 1: Table S1). At the time of analysis, these represented the highest quality assemblies with known, non-redundant geographic origin from either North America or Europe. I also included a ninth, outgroup genome that was sequenced from *A. phagocytophilum* infecting a roe deer (representing the roe-deer specialist ecotype). All genomes with the exception of the host-generalist from France and the roe deer-specialist outgroup comprised a single, linearized chromosome.

### Clustering and phylogenetic analyses

To confirm the phylogenetic placement of these genomes within previously established *A. phagocytophilum* diversity, I utilized an additional, published dataset that comprised partial sequences from seven housekeeping genes (*atpA*, *dnaN*, *fumC*, *glyA*, *mdh, pheS* and *sucA*; GenBank accession numbers: KF242733-KF245413). These gene regions were developed as part of a multi-locus sequence typing (MLST) study, and were sequenced using *A. phagocytophilum* DNA isolated from 17 different European mammalian hosts (both wild and domestic), as well as *I. ricinus* ticks [6]. Also included in this study were 11 samples that originated from the USA. Collectively, these samples were found to represent three discrete genetic groups, which were hypothesized to have independent enzootic cycles among mammalian hosts [6].

Prior to my analyses, for each sample I concatenated the seven gene regions (2877 nucleotides in total), following which I removed all sequences with ≥ 1 ambiguous nucleotides. I also removed all but one of any redundant haplotype. This left 92 unique samples (haplotypes). To these, I added orthologous, concatenated sequences from each of the nine focal genomes (see above); these gene regions were located using a BLAST search [33], with each genome as a translated nucleotide database (‘tblastn’, *e* = 1e-10). Consensus gene sequences from the MLST dataset were used for the BLAST queries. As these are highly conserved gene regions, each BLAST comparison yielded a single, high-confidence match between the reference genome and the query sequence.

With this dataset of 92 unique MLST samples plus the orthologous data from the nine focal genomes, I first examined sample clustering using a nonparametric principal component analysis (PCA). Prior to this analysis, I removed the 10 unique sequences representing the burrowing-mammal specialist ecotype [6]. The PCA was conducted with the R package Adegenet version 2.1.8 [34, 35], as implemented in R v.4.1.2 [36]. I also used R to visualize the relationship between the first and second principal components (PCs).

I next used the full dataset to reconstruct phylogenetic relationships between the samples using the approximately maximum likelihood (ML) approach in IQ-tree v.1.6.12 [37]. ModelFinder [38], as integrated within IQ-tree, was used to determine the best model for the first, second and third position of each of the seven gene regions independently [39]. I calculated support values from 10,000 ultrafast bootstrap (UFBoot) replicates [40], as well as 10,000 replicates of the Shimodaira-Hasegawa-like approximate likelihood ratio test (SH-aLRT [41–43]). The resulting phylogenetic tree was visualized and edited with the program FigTree v.1.4.4 [44].

### Identification of host-specific and core genes

To identify genes that are differentially expressed during specific host interactions, I obtained from the authors previously generated and published gene expression data from *A. phagocytophilum* replicating in either human HL-60 cell lines or *Ixodes scapularis* ISE6 cell lines [45]. These data were generated by hybridizing *A. phagocytophilum* (Ap-ha) messenger RNA (mRNA) to a custom-designed tiling microarray that represented the entire *A. phagocytophilum* genome. The data I received had been quality checked and normalized as previously described [45]. To determine a relative expression value for each gene in each sample replicate, I summed the hybridization values for each probe corresponding to a specific gene, then divided this sum by the total number of probes that overlapped the gene.

As the datasets represented multiple different time points for each cell type (Additional file 1: Table S2 [45]), I examined two broad categories of expression data. The first of these consisted of eight datasets showing *A. phagocytophilum* gene expression patterns when this bacterium was infecting HL-60 cells. The second consisted of seven datasets showing *A. phagocytophilum* gene expression patterns when infecting ISE6 cells. I calculated the mean expression level per cell type (HL-60 vs ISE6) for each gene, then determined the ratio of mean expression in HL-60 cells over mean expression in ISE6 cells. Using the expression values from each dataset for each cell type, I also performed a two-sample t-test to look for statistically significant differences in expression levels for each gene between the two cell types. Any gene with both HL-60/ISE6 ratio > 2.0 and statistically significant at *P* < 0.05 in the t-test was classified as a ‘reservoir-upregulated’ gene. Conversely, any gene with HL-60/ISE6 ratio < 0.5 and statistically significant at *P* < 0.05 in the t-test was classified as a ‘vector-upregulated’ gene. These ratios (> 2.0 and < 0.5) correspond to a twofold or greater difference in expression level between *A. phagocytophilum* replication in HL-60 versus ISE6, respectively. For a control group, I defined a third category from those genes without a statistically significant t-test at *P* < 0.05 and which had a HL-60/ISE6 ratio > 0.9 and < 1.1; these genes were classified as ‘core’ genes, as they did not appear to vary in expression level in response to the host environment.

### Location and organization of orthologous gene sequences

To compare patterns of gene evolution and potential divergence between North American and European *A. phagocytophilum* populations, from each of the nine genomes (Fig. 1a; Additional file 1: Table S1) I located the gene orthologues of my identified vector-upregulated genes, reservoir-upregulated genes and core genes (see section Identification of host-specific and core genes). To locate these genes, I used protein query sequences taken from the annotated genome assembly of *A. phagocytophilum* strain HGE-1 (NCBI-GenBank accessions: APHH01000001.1, APHH01000002.1). With these query protein sequences, I conducted a BLAST search [33], using each genome as a translated nucleotide database (‘tblastn’, *e* = 1e-10). Then, for each gene, I used a custom Perl wrapper integrating Samtools v.1.14 [46] to combine the top BLAST matches for each of the nine focal genomes into a single, gene-specific FASTA file. Finally, I checked each gene’s FASTA file by eye in SeaView v.5.0.4 for proper alignment, sequence redundancies and fragmented sequences [47]. Any insertions/deletions (INDELs) were removed along with any immediately adjacent, ambiguous amino acids sites. Fragmented sequences for any sample were concatenated.

### Divergence time estimates

To estimate divergence times between the samples, I concatenated the 125 core gene alignments into single, sample-specific sequences. I then used IQtree v.1.6.12 [37] to infer a maximum-likelihood tree from this sequence alignment, with the best-fit model automatically selected separately for the first, second and third codon positions (partitioned analysis). With my concatenated dataset and the tree produced from IQtree, I used the RelTime-ML function [48, 49] in the program MEGA v.11.0.10 [50, 51] to produce absolute estimates of divergence time between each sample. To calibrate my time estimates, I used the Tao method [52] to set minimum and maximum time boundaries based on the previously calculated divergence time of 2970 years (95% highest probability density [HPD] 454–7,240 years) between the European roe deer-specialist ecotype and the European host-generalist ecotype [53]. A log-normal distribution was used for this calibration point, with an offset of 454 years and a standard deviation of 4.19. For modeling rates of evolution, I used a gamma distributed general time reversible (GTR) model with a proportion of invariant sites (GTR + I [54]).

### Genetic diversity estimates

To examine relative nucleotide diversity levels in European and North American *A. phagocytophilum,* I calculated two summary statistics of diversity for each gene. The first was the average number of nucleotide differences observed between samples per site (π [55]). The second was the number of segregating sites per locus, corrected for sequence length (θ_W_ [55, 56]). Both π and θ_W_ were calculated following Nei (see Eqs. 10.5 and 10.3 [55]). For both of these statistics, separate estimates for synonymous and non-synonymous sites were calculated, and gene averages were determined for vector-upregulated, reservoir-upregulated and core genes independently. A custom Perl script was used to perform these diversity calculations, with code modified from the program Polymorphorama to determine whether a segregating site was non-synonymous or synonymous [57], as well as the number of potential non-synonymous and synonymous sites in a sequence. A paired t-test, as implemented in R v.4.1.2 [36], was used to determine statistically significant differences for all diversity measures between North American and European genes (assessing vector-upregulated, reservoir-upregulated and core genes separately). I used the Kruskal–Wallis rank sum test [58] to examine statistical differences within a geographic region for each diversity measure between each of the three gene categories. This test was also implemented in R.

### Determination of derived alleles

Within the gene alignments for each category (vector-upregulated, reservoir-upregulated and core genes), if one of two biallelic nucleotides observed in any of the eight focal samples (4 North American Ap-ha variant samples and 4 European host-generalist ecotype samples) was also observed at the orthologous roe deer-specialist ecotype site (outgroup), it was considered to be the ancestral allele. Accordingly, the alternative allele was classified as the derived allele. A site was considered fixed within a population if all four genomes for the focal geographic regions (North America vs Europe) harbored the same allele. Non-synonymous and synonymous sites were examined separately. To normalize the number of observed, fixed, derived sites by the amount of sequence analyzed per gene, I divided the observed counts of non-synonymous or synonymous, fixed, derived alleles in each population by the total number of potential non-synonymous or synonymous sites in that sequence (i.e. K_a_ & K_s_, respectively). These estimates were conducted with a custom Perl script utilizing code modified from the program Polymorphorama [57]. The statistical methods used to compare the number of derived alleles observed between North American and European genes were the same as those described in section Genetic diversity estimates.

### Population divergence estimates

To examine divergence between the European and North American *A. phagocytophilum* within each of the three gene categories, I calculated the average number of nucleotide differences per locus (d_XY_; see Eq. 10.20 [55]). Separate estimates for synonymous and non-synonymous sites were calculated, and gene averages were determined for vector-upregulated, reservoir-upregulated and core genes separately. I also used my estimates of within-population nucleotide differentiation per site (π [55]) to calculate the average number of net nucleotide substitutions per site for each gene (d_A_; see Eq. 10.21 [55]). A custom Perl script was used to perform these calculations. Averages and standard deviations for each of the three gene categories were determined using R v.4.1.2 [36]. The Kruskal–Wallis rank sum test [58] was used to examine statistical differences for each divergence measure between each of the three gene categories.

## Results

### Population relationships

In my PCA using all unique MLST haplotypes, the samples previously assigned to the roe deer-specialist ecotype were distinct from the other samples along the first PC (PC1; Fig. 1b). This PC represented 9.82% of the observed genetic variance. Along PC2, representing 3.97% of the observed genetic variance, the European-derived samples previously assigned to the host-generalist ecotype formed a broad cluster that was distinct from the Ap-ha variant samples from North America. The data representing the nine genomes were each found within their expected clusters.

As with the PCA, my ML phylogenetic analysis showed that the roe deer-specialist ecotype samples were diverged from the European-derived, host-generalist ecotype samples, as well as from the North American Ap-ha genome samples. The host-generalist ecotype formed a unique clade, with the North American samples comprising a subclade within it (Fig. 1c; Additional file 2: Figure S1). All the major nodes distinguishing different ecotypes and/or geographic regions had ultrafast bootstrap values ≥ 95% and SH-aLRT values ≥ 85%, both of which indicate estimated confidence levels > 95% [59].

### Patterns of gene expression

The analysis identified 61 genes that were upregulated by two-fold or greater in *A. phagocytophilum* when this bacterium was infecting ISE6 cells, compared to when it was infecting HL-60 cells (Additional file 1: Table S2). After excluding any genes for which there was no regions of overlap for all nine genomic samples, I was left with 52 gene regions for subsequent analyses. These I characterized as ‘vector-upregulated’ genes. I also found 112 genes that were upregulated by twofold or greater in *A. phagocytophilum* when this bacterium was infecting HL-60 cells, compared to when it was infecting ISE6 cells. This list included 35 identified p44 genes; I did not use these p44 genes in subsequent analyses due to challenges in confidently identifying orthologous between samples. I also removed any genes with excessive missing data. This left me with 67 gene regions designated as ‘reservoir-upregulated’. Finally, I found 155 genes that were similarly expressed regardless of the host environment. After alignment and manual assessment, I was left with 125 of these ‘core’ genes for subsequent analysis. These genes were assumed to be expressed independent of host environment and were used as a control when examining patterns of gene evolution and divergence in vector- or reservoir-upregulated genes.

### Divergence times

Using the identified ‘core’ gene alignments (see section Patterns of gene expression ) and a ML approach, I estimated that the assessed genomes from the European host-generalist ecotype and those from North America last shared a common ancestor approximately 1547 years ago (95% confidence interval [CI] 1451—1650 years; Additional file 2: Figure S2). However, the North America Ap-ha samples were estimated to have shared a common ancestor approximately 44 years ago (95% CI 35—56 years; Additional file 2: Figure S2).

### Genetic diversity and derived alleles

**Fig. 2:**
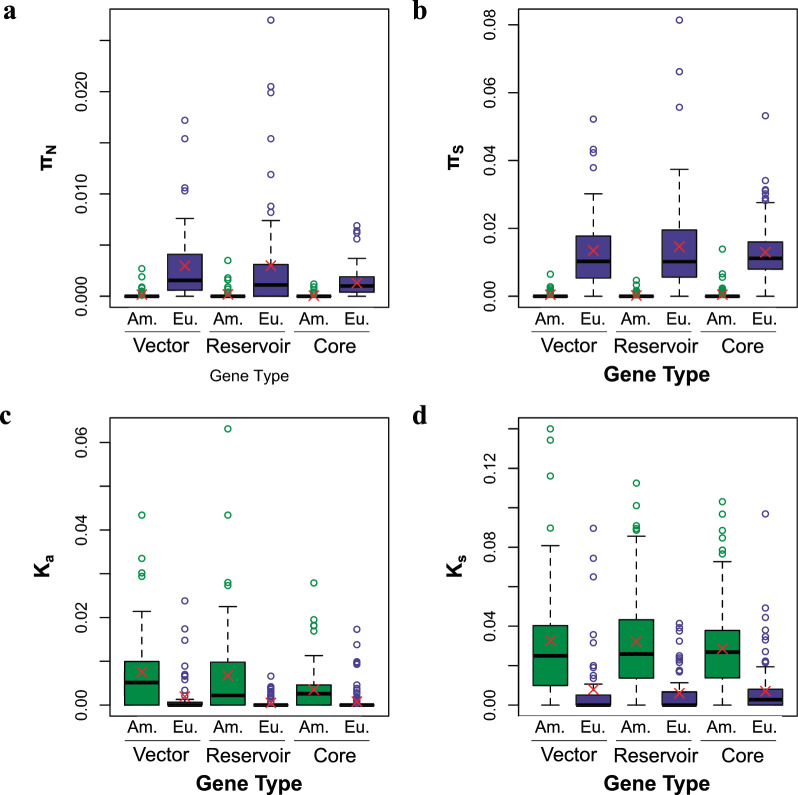
Boxplots showing variation in genetic diversity and derived divergence. **a** Genetic diversity at non-synonymous sites (π_N_). **b** Genetic diversity at synonymous sites (π_S_). **c** Number of non-synonymous, fixed, derived mutations per site (K_a_). **d** Number of synonymous, fixed, derived mutations per site (K_s_). Median values are indicated by thick, black horizontal lines, and mean values are indicated by red ‘X’s. Outlying datapoints are indicated by open circles. Data is divided by gene type (vector-upregulated, reservoir-upregulated and core genes, respectively), and by geographic region (America or Europe). North American data are represented in green, and European data are represented in purple. Am., North America; Eu., Europe

For both diversity measures at non-synonymous sites (π and θ_W_), the European samples harbored significantly more genetic diversity for all three gene categories, compared to the North American samples (Table 2; Fig. 2a; Additional file 1: Table S3; Additional file 2: Figure S3). Among the gene categories for the European genomes, both vector- and reservoir-upregulated genes harbored significantly more genetic diversity, compared to core genes. There was no statistically significant difference in the genetic diversity levels observed in the three gene categories for the North American genomic data.

For synonymous sites, again the European samples harbored significantly more genetic diversity compared to the North American samples for all three gene categories (Table 2; Fig. 2b; Additional file 1: Table S3; Additional file 2: Figure S3). However, there was no significant differences between the gene categories for either diversity measure in either geographic region.

On average, the North American *A. phagocytophilum* genomes harbored statistically more derived alleles per site than the European *A. phagocytophilum* genomes for all three gene categories, at both non-synonymous (K_a_) and synonymous sites (K_s_) (Tables 1, 2; Fig. 2c, d; Additional file 1: Table S4). There were no significant differences between gene categories for either geographic region in either site class in the number of observed, derived alleles.

**Table 1:** Average estimates of genetic diversity, and derived alleles at non-synonymous sites for both the North American (Ap-ha) and European (host-generalist ecotype) genomes

Genetic diversity estimates and derived alleles estimate^a^	Vector-upregulated genes (*n* = 52)		Reservoir-upregulated genes (*n* = 67)		Core genes (*n* = 125)		Kruskal–Wallis rank sum test results (*df* = 2)^d^
	*μ* (SD)^b^	Paired t-test results (*df* = 51)^c^	*μ* (SD)^b^	Paired t-test result (*df* = 66)^c^	*μ* (SD)^b^	Paired t-test results (*df* = 124)^c^	
π_N_ America	0.00016 (0.00048)	*t* = − 5.574 *P* < 0.0001*	0.00019 (0.00054)	*t* = − 4.545 *P* < 0.0001*	0.00006 (0.00018)	*t* = − 10.610 *P* < 0.0001*	*χ*^2^ = 3.955 *P* = 0.1384#
π_N_ Europe	0.00298a (0.00370)		0.00300a (0.00525)		0.00130b (0.00130)		*χ*^2^ = 6.741 *P* = 0.0344
θ_W_ America	0.00031 (0.00092)	*t* = − 5.687 *P* < 0.0001*	0.00035 (0.00102)	*t* = − 4.596 *P* < 0.0001*	0.00010 (0.00033)	*t* = − 10.534 *P* < 0.0001*	*χ*^2^ = 4.021 *P* = 0.1339
θ_W_ Europe	0.00518a (0.00631)		0.00525a (0.00907)		0.00230b (0.00231)		*χ P*^2^ = 6.204 *P* = 0.0450#
Derived sites America (K_a_)	0.00754 (0.00957)	*t* = 3.832 *P* = 0.0003*	0.00678 (0.01112)	*t* = 4.742 *P* < 0.0001*	0.00350 (0.00439)	*t* = 6.304 *P* < 0.0001*	*χ*^2^ = 4.517 *P* = 0.1045
Derived sites Europe (K_a_)	0.00194 (0.00478)		0.00050 (0.00126)		0.00079 (0.00253)		*χ*^2^ = 2.534 *P* = 0.2816

*SD* Standard deviation

^a^π_N_, Average number of nucleotide differences observed between samples per non-synonymous site; θ_W_, number of segregating sites per locus, corrected for sequence length; K_a_, number of fixed, derived alleles in each population divided by the total number of potential non-synonymous sites in that sequence

^b^Averages (*μ*) followed by different lowercase letters indicate which comparisons were significantly different from one another

^c^*P*-values marked with asterisk (*) indicate comparisons that were significantly different between geographic regions at* P* < 0.05 according to the paired *t*-test results

^d^*P*-values marked with a hash sign (#) indicate comparisons that were significantly different between geographic regions at* P* < 0.05 according to the Kruskal-Wallis rank sum test results

**Table 2:** Average estimates of genetic diversity, and derived alleles at synonymous sites for both the North American (Ap-ha) and European (host-generalist ecotype) genomes

Genetic diversity estimates and derived alleles estimate^a^	Vector-upregulated genes (*n* = 52)		Reservoir-upregulated genes (*n* = 67)		Core genes (*n* = 125)		Kruskal–Wallis Rank sum test results (*df* = 2)
	*μ* (SD)	Paired t-test results (*df* = 51)^b^	*μ* (SD)	Paired t-test result (*df* = 66)^b^	*μ* (SD)	Paired t-test result (*df* = 124)^b^	
π_S_ America	0.00044 (0.00111)	*t* = − 7.797 *P* < 0.0001*	0.00022 (0.00077)	*t* = − 7.994 *P* < 0.0001*	0.00047 (0.00153)	*t* = − 17.562 *P* < 0.0001*	*χ*^2^ = 3.371 *P* = 0.1853
π_S_ Europe	0.01347 (0.01203)		0.01463 (0.01494)		0.01295 (0.00803)		*χ*^2^ = 0.802 *P* = 0.6698
θ_W_ America	0.00070 (0.00169)	*t* = − 7.811 *P* < 0.0001*	0.00038 (0.00122)	*t* = − 8.006 *P* < 0.0001*	0.00083 (0.00268)	*t* = − 17.766 *P* < 0.0001*	*χ*^2^ = 3.250 *P* = 0.1969
θ_W_ Europe	0.02305 (0.02063)		0.02543 (0.02592)		0.02251 (0.01377)		*χ*^2^ = 1.070 *P* = 0.5856
Derived Sites America (K_s_)	0.03266 (0.03392)	*t* = 5.204 *P* < 0.0001*	0.03227 (0.02738)	*t* = 7.627 *P* < 0.0001*	0.02855 (0.02144)	*t* = 9.410 *P* < 0.0001*	*χ*^2^ = 0.415 *P* = 0.8128
Derived Sites Europe (K_s_)	0.00791 (0.01895)		0.00597 (0.01044)		0.00702 (0.01276)		*χ*^2^ = 3.514 *P* = 0.1725

^a^π_S_, Average number of nucleotide differences observed between samples per synonymous site; θ_W_, number of segregating sites per locus, corrected for sequence length; K_s_ number of fixed, derived alleles in each population divided by the total number of potential synonymous sites in that sequence

^b^*P*-values marked with asterisk (*) indicate comparisons that were significantly different between geographic regions at* P* < 0.05 according to the paired *t*-test results

### Population divergence

**Fig. 3:**
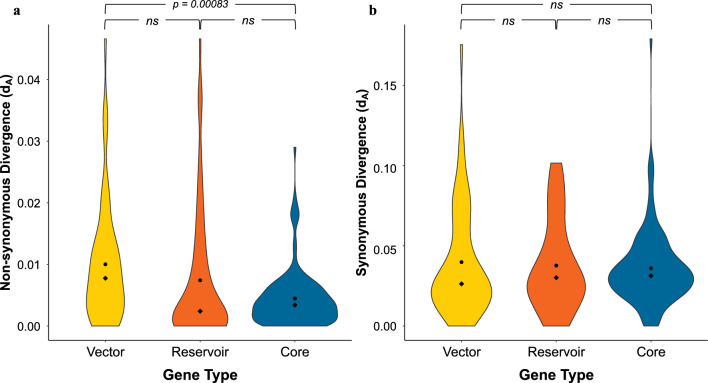
Violin plots showing the distribution of per-gene genetic diversity estimates per site corrected for polymorphism (d_A_). Estimates are given for each gene type: vector-upregulated (yellow), reservoir-upregulated (orange) or core (blue) genes. **a** The distribution of d_A_ values for non-synonymous sites, **b** the distribution of d_A_ values for synonymous sites. All pairwise comparisons were non-significant except that between vector-upregulated genes and core genes for non-synonymous sites, which was significant at *P* = 0.0009 (Kruskal–Wallis rank sum test: *χ*^2^ = 14.10,* df* = 2). ns, Non-significant

For non-synonymous d_XY_, vector-upregulated genes and reservoir-upregulated genes were significantly more diverged than core genes (Table 3; Additional file 1: Table S5; Additional file 2: Figure S4). When I accounted for genetic polymorphism in my divergence calculation (d_A_), reservoir-upregulated genes were not significantly different from either vector-upregulated genes or core genes (Table 3; Fig. 3a; Additional file 1: Table S5; Additional file 2: Figure S4). However, vector-upregulated genes were significantly more diverged than core genes. For synonymous sites, there were no significant difference between the three gene classes for either measure of divergence (d_XY_ & d_A_; Table 3; Fig. 3b; Additional file 1: Table S5; Additional file 2: Figure S4b).

**Table 3:** Average estimates of non-synonymous and synonymous genetic divergence and genetic divergence corrected for polymorphism levels between North American (Ap-ha) and European (host-generalist ecotype) genomes

Measures of genetic divergence^a^	Vector-upregulated genes (*n* = 52)	Reservoir-upregulated genes (*n* = 67)	Core genes (*n* = 125)	Kruskal–Wallis rank sum test results (*df* = 2)^c^
	*μ* (SD)^b^	*μ* (SD)^b^	*μ* (SD)^b^	
d_XY_ non-synonymous	0.01158a (0.01119)	0.00900a (0.01314)	0.00514b (0.00508)	*χ*^2^ = 14.60 *P* = 0.0007#
d_A_ non-synonymous	0.01001a (0.00991)	0.00741a,b (0.01064)	0.00446b (0.00475)	*χ*^2^ = 14.10 *P* = 0.0009#
d_XY_ synonymous	0.04678 (0.03870)	0.04509 (0.03392)	0.04266 (0.02599)	*χ*^2^ = 0.404 *P* = 0.8170
d_A_ synonymous	0.03982 (0.03482)	0.03767 (0.02827)	0.03595 (0.02362)	*χ*^2^ = 0.433 *P* = 0.8054

^a^d_XY_, Average estimate of genetic divergence (number of nucleotide differences per locus); d_A_, genetic divergence corrected for polymorphism level (average number of net nucleotide substitutions per site for each gene)

^b^Averages (*μ*) followed by different lowercase letters indicate which comparisons were significantly different from one another

^c^*P*-values marked with a hash sign (#) indicate comparisons that were significantly different between geographic regions at* P* < 0.05 according to the Kruskal-Wallis rank sum test results

As gene expression levels may influence patterns of divergence (e.g. [60, 61]), I also compared overall gene expression levels (average expression in both HL-60 and ISE6 cells, combined; Additional file 1: Table S2) to divergence after correction for genetic polymorphism (d_A_). This analysis showed a small but significant negative relationship between expression level and gene divergence in core genes (*F*_1,123_ = 9.3, *P* = 0.0028; Additional file 2: Figure. S5). However, for both vector-upregulated and reservoir-upregulated genes, there was no significant relationship between expression and divergence (vector-upregulated genes: *F*_1,50_ = 0.02191, *P* = 0.8829; reservoir-upregulated genes: *F*_1,65_ = 1.017, *P* = 0.3169).

## Discussion

The nested phylogenetic relationship of the North American Ap-ha variant within the diversity of the European host-generalist ecotype, plus Ap-ha’s recent radiation, low genetic diversity and high number of derived alleles, all suggest a North American introduction from Europe, likely with a corresponding founder event that resulted in a genetic bottleneck [23]. Although speculative, one clear possibility is that *A. phagocytophilum* may have been introduced via infected domestic animals brought to the North American continent from Europe [62]. *Anaplasma phagocytophilum* may have also been introduced to North America through infected birds or ticks [62].

Alternatively, it may be that the observations revealed here are the result of a substantial bottleneck or selective sweep in North American *A. phagocytophilum* [23], independent of a founding event associated with this lineage’s introduction to the continent. However, while the complex transmission dynamics of *A. phagocytophilum* mean that they likely go through frequent, local bottlenecks, variations in host demography and additional ecological heterogeneity at a continental scale make a bottleneck or selective sweep affecting a widespread population unlikely [63].

My observation that samples of the North American Ap-ha variant harbor significantly more derived alleles compared to the European host-generalist ecotype suggest that observed genetic differences within the *A. phagocytophilum* of these two locations are largely driven by evolutionary changes that occurred in the North American lineage. One particularly appealing hypothesis associated with this observation is that adaptation to novel vector and/or reservoir species in North America may have accelerated genome divergence. In support of this hypothesis, vector-upregulated genes showed increased levels of divergence between North American and European genomes, relative to core genes (Table 3; Fig. 3; Additional file 2: Figure S4). Further analyses will be needed to assess specific patterns of gene evolution in relation to vector-adaption in North American *A. phagocytophilum*.

Regarding the question of pathogenicity disparities between the America Ap-ha variant and the European host-generalist ecotype, this study supports the hypothesis that genetic differences between the two populations could be a contributing factor. It is likely that during its evolution in North America, changes in *A. phagocytophilum* occurred that may have altered its virulence and/or host densities. Changes in host environment, especially in the context of a new invasion, can have significant effects on pathogen virulence [64, 65]. While work examining potential markers associated with pathogenicity have not revealed any consistent differences (e.g. [15]), such research is still in its early stages, and future studies may reveal genetic changes in Ap-ha that contribute to its greater pathogenicity in humans relative to other populations of *A. phagocytophilum.*

## Conclusions

The results of this study suggest that the North American Ap-ha variant of *A. phagocytophilum* is derived from European *A. phagocytophilum.* Support for this conclusion comes from the observation that Ap-ha diversity is phylogenetically nested within the diversity of the host-generalist ecotype, as well as the low genetic diversity of Ap-ha, the high number of derived alleles it harbors, and its recent radiation. This work has implications for understanding variation in pathogenicity between the North American and European populations of *A. phagocytophilum*. A better understanding of the forces that have resulted in divergent patterns of pathogenicity between North American and European *A. phagocytophilum* may help inform public health initiatives aimed at reducing the negative impacts of this bacterial pathogen [19].

### Supplementary Information

**Additional file 1: Table S1. **Genome sample information. **Table S2. **Relative values for *A. phagocytophilum* genes when this bacterium is replicating in either human cell lines (HL-60) or *Ixodes scapularis* cell lines (ISE6). **Table S3. **Gene type (vector-upregulated, reservoir-upregulated, or core), gene IDs (from *A. phagocytophilum* strain HGE-1) and the total number of nucleotides (sites) analyzed. **Table S4. **Gene type (vector-upregulated, reservoir-upregulated or core), gene IDs (from *A. phagocytophilum* strain HGE-1), and protein ID names (columns 1-3). **Table S5. **Gene specific measures of divergence.

**Additional file 2: Figure S1. **Phylogeny subset showing just the host-generalist ecotype samples (blue) and the American Ap-ha variants samples (green). **Figure S2. **Divergence time estimates using the RelTime-ML function [48, 49], in the program MEGA v.11.0.10 [50, 51]. **Figure S3. **Boxplots showing variation in the number of segregating sites per locus, corrected for sequence length θ_W_.** a** θ_W_ at non-synonymous sites,** b** θ_W_ at synonymous sites. **Figure S4. **Violin plots showing the distribution of per-gene genetic diversity estimates per site ( d_XY_).** a** Distribution of d_XY_ values for non-synonymous sites,** b** distribution of d_XY_ values for synonymous sites. **Figure S5. **Correlations between overall gene expression levels (average expression in both HL-60 and ISE6 cells, combined; Additional file: Table S2) and divergence after correction for genetic polymorphism (d_A_).

## Data Availability

All data used in this study is publicly available in conjunction with prior studies. Appropriate data identifiers (e.g. accession numbers) are given either in the Methods section or Additional file 1: Table S1. Custom Perl scripts used in the analyses are available from the author upon request.

## Abbreviations

Am.: North America
CI: Confidence interval
Eu.: Europe
GTR: Generalized time reversible model
ML: Maximum likelihood
PC: Principal component
PCA: Principal component analysis
