# Supplementary material for: Genomic analyses indicate the North American Ap-ha variant of the tick-vectored bacterium Anaplasma phagocytophilum was introduced from Europe
Source: Parasit Vectors. 2023 Aug 28;16:301. doi: 10.1186/s13071-023-05914-x (PMC10463431; doi:10.1186/s13071-023-05914-x)
Supplement: Supplementary file 2 — Additional file 2: Figure S1. Phylogeny subset showing just the host-generalist ecotype samples (blue) and the American Ap-ha variants samples (green). Figure S2. Divergence time estimates using the RelTime-ML function [48, 49], in the program MEGA v.11.0.10 [50, 51]. Figure S3. Boxplots showing variation in the number of segregating sites per locus, corrected for sequence length θW. a θW at non-synonymous sites, b θW at synonymous sites. Figure S4. Violin plots showing the distribution of per-gene genetic diversity estimates per site ( dXY). a Distribution of dXY values for non-synonymous sites, b distribution of dXY values for synonymous sites. Figure S5. Correlations between overall gene expression levels (average expression in both HL-60 and ISE6 cells, combined; Additional file: Table S2) and divergence after correction for genetic polymorphism (dA). [file 13071_2023_5914_MOESM2_ESM.docx]

Additional file for:

**Genomic analyses indicate the North American Ap-ha variant of the tick-vectored bacterium *Anaplasma phagocytophilum* was introduced from Europe**

Matthew L. Aardema

See Additional file 1

**Additional file 1, Table S1.** Genome Sample Information. Column one, ‘Broad geographic origin’, indicates whether the sample was isolated from North America or Europe. Column two, ‘Population’, indicates whether the sample was determined to belong to the Ap-ha variant (North America) or the host-generalist ecotype (Europe). Also, shown is the outgroup sample determined to belong to the roe deer-specialist ecotype. Sample IDs (column three) are the unique identifiers used in the paper text, tables, and figures. The National Institute of Health’s (NIH) National Center for Biotechnology Information (NCBI) unique assembly identifier (NCBI Accession) is given in the fourth column. Column five gives the mammalian reservoir/host from which the *A. phagocytophilum* was isolated for genome sequencing. Column six indicates how fragmented the genome assembly was at the time of use.

**Additional file 1, Table S2.** Relative values for *A. phagocytophilum* genes when this bacterium is replicating in either human cell lines (HL-60) or *Ixodes scapularis* cell lines (ISE6). Relative expression values for each gene in each sample replicate were calculated by summing the hybridization values for each probe corresponding to a specific gene, then divided this by the total number of probes that overlapped the gene. The raw data used for these calculations was previously generated and published as described in Nelson et al., (2020). A two-sample t-test was used to look for statistically significant differences in expression levels between the two groups. I also calculated the means of the expression levels for each gene type, then determined the ratio of mean expression in HL-60 cells over mean expression in ISE6 cells (column 22). Any gene that was statistically significant at p < 0.05 in the t-test and which had a HL-60/ISE6 ratio greater than 2.0 was classified as a ‘reservoir-upregulated’ gene. Conversely, any gene that was statistically significant at p < 0.05 in the t-test and which had a HL-60/ISE6 ratio less than 0.5 was classified as a ‘vector-upregulated’ gene. I defined a third category from those genes without a statistically significant t-test at p < 0.05, and which had a HL-60/ISE6 ratio between 0.9 and 1.1. These genes were classified as ‘core’ genes. Genes that were not placed into one of these three categories are not shown.

**Additional file 1, Table S3.** Gene type (vector-upregulated, reservoir-upregulated, or core), Gene IDs (from *A. phagocytophilum* strain HGE-1), and the total number of nucleotides (sites) analyzed (columns 1-3). Columns 6-9 are diversity statistics (π & θ_W_) for North American samples (Ap-ha), calculated separately for non-synonymous and synonymous sites. Columns 10-13 are diversity statistics (π & θ_W_) for European samples (host-generalist ecotype), calculated separately for non-synonymous and synonymous sites.

**Additional file 1, Table S4.** Gene type (vector-upregulated, reservoir-upregulated, or core), Gene IDs (from *A. phagocytophilum* strain HGE-1), and protein ID names (columns 1-3). For North American samples, the number of fixed, substitutions per site (K) are given for non-synonymous sites (K_a_) and synonymous sites (K_s_).

**Additional file 1, Table S5.** Gene specific measures of divergence. Gene type (vector-upregulated, reservoir-upregulated, or core) and Gene IDs (from *A. phagocytophilum* strain HGE-1) are given in columns one and two. The average number of nucleotide differences per locus (d_XY_) is given for non-synonymous (column 3) and synonymous (column 4) site separately. Also given are the average number of net nucleotide substitutions per site for each gene (dA) for non-synonymous (column 5) and synonymous (column 6) sites separately.

**Figure S1.** Phylogeny subset showing just the host-generalist ecotype samples (blue) and the American Ap-ha variants samples (green). The naming of the nodes represented by genome data are as in Table S1. The red numbers next to representative nodes indicate support values from 10,000 ultrafast bootstrap (UFBoot) replicates (Hoang et al., 2018), and 10,000 replicates of the Shimodaira–Hasegawa-like approximate likelihood ratio test (SH-aLRT; Kishino & Hasegawa, 1989; Shimodaira & Hasegawa, 1999; Guindon et al., 2010). This tree was visualized and edited with the program FigTree v.1.4.4 (Rambaut, 2018). The insert shows the full tree and is identical to Figure 1c.

**Figure S2.** Divergence time estimates using the RelTime-ML function (Tamura et al., 2012; Tamura et al., 2018), in the program MEGA v.11.0.10 (Stecher et al., 2020; Tamura et al., 2021). I used the Tao method (Tao et al., 2020) to set minimum and maximum time boundaries based on the previously calculated divergence time of 2,970 years (95% HPD 454 - 7,240 years) between the European roe deer-specialist ecotype and the European host-generalist ecotype (Aardema et al., 2022). A log-normal distribution was used for this calibration point, with an offset of 454 years and a standard deviation of 4.19. For modeling rates of evolution, I used a gamma distributed general time reversable model with a proportion of invariant sites (GTR + I; Nei & Kumar, 2000). Blue nodes represent the North American Ap-ha samples, green nodes represent European host-generalist ecotype samples and the red node represents the roe deer-specialist ecotype sample (outgroup). The gray bars and values in parentheses indicate confidence intervals on the divergence estimates.

**Figure S3.** Boxplots showing variation in the number of segregating sites per locus, corrected for sequence length θ_W_. (a) θ_W_ at non-synonymous sites. (b) θ_W_ at synonymous sites. Median values are indicated by thick, black horizontal lines and mean values are indicated by the red ‘X’s. Outlying datapoints are indicated by open circles. Data is divided by gene type (vector-upregulated, reservoir-upregulated, and core genes), and by geographic region (America [Am.] or Europe [Eu.]).

**Additional Figure S4.** Violin plots showing the distribution of per-gene genetic diversity estimates per site, d_XY_. Estimates are given for each gene type; vector-upregulated, reservoir-upregulated, or core genes (a) The distribution of d_XY_ values for non-synonymous sites. (b) The distribution of d_XY_ values for synonymous sites. All pairwise comparisons were non-significant (*ns*) except those between vector-upregulated genes and core genes for non-synonymous sites, and between reservoir-upregulated genes and core genes for non-synonymous sites (Kruskal-Wallis rank sum test: χ2 = 14.6, df = 2, p-value = 0.0007).

**Figure S5.** Correlations between overall gene expression levels (average expression in both HL-60 and ISE6 cells, combined; Table S2) and divergence after correction for genetic polymorphism (d_A_). Vector-upregulated (yellow), reservoir-upregulated (orange) and core (blue) genes were examined separately. The coefficient of determination (R^2^) for each comparison is given next to the corresponding regression lines. Only the correlation between expression and divergence for the core genes was found to be statistically significant (vector-upregulated genes: F_1,50_ = 0.02191, p = 0.8829; reservoir-upregulated genes F_1,65_ = 1.017, p = 0.3169; core genes F_1,123_ = 9.3 p = 0.0028).
